# Iron-containing mesoporous aluminosilicate catalyzed direct alkenylation of phenols: Facile synthesis of 1,1-diarylalkenes

**DOI:** 10.3762/bjoc.9.6

**Published:** 2013-01-09

**Authors:** Satyajit Haldar, Subratanath Koner

**Affiliations:** 1 Department of Chemistry, Jadavpur University, Kolkata 700032, India

**Keywords:** 1,1-diarylalkenes, heterogeneous catalysis, Fe-Al-MCM-41

## Abstract

The addition of phenols to aryl-substituted alkynes to form 1,1-diarylalkenes was carried out by using the Fe-Al-MCM-41 catalyst. The catalyst showed remarkable improvement in time and yield in comparison to other solid catalysts. The heterogeneous catalyst can be reused at least three times without a significant loss in activity.

## Introduction

**Figure 1:**
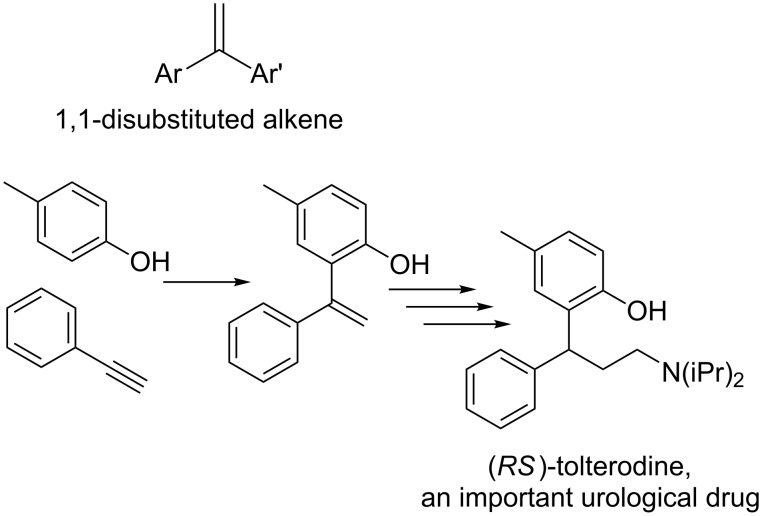
(*RS*)-Tolterodine, an important urological drug.

The direct vinylation of phenols has received considerable attention by synthetic organic chemists for a long time. The resulting 1,1-disubstituted alkene derivatives (Figure 1) are widely used as key starting materials in the synthesis of fine chemicals [1–7], polymers [8–14], and pharmaceuticals [15–17]. Furthermore, a variety of available reactions to functionalize the double bond, such as reductive (hydrogenation, hydrosilylation, etc.), oxidative (epoxidation, halogenations, dihydroxylation, etc.) or cycloaddition transformations, encourage such vinylation process as an attractive primary tool in organic synthesis. Thus, the development of simple methods concerning such vinylation reactions of phenols always remains an important process.

Mizoroki–Heck-type reaction could be considered as an efficient procedure for the synthesis of such vinylated phenols [18–19]. There are a number of reports available in the literature that involve Pd-catalyzed cross coupling reactions of various aryl halides with different olefins. However, the major drawback of the Mizoroki–Heck-type reaction in the synthesis of 1,1-disubstituted olefins rests on its poor selectivity toward the formation of α-products [20–23]. In general, the reaction shows β-selectivity, and there are only a few reports available concerned with α-substituted products [24–26]. Sabarre and Love reported a one-pot rhodium-catalyzed alkyne hydrothiolation followed by a nickel-catalyzed Kumada-type cross coupling with Grignard reagents to afford 1,1-disubstituted olefins [27]. Alternatively, Friedel–Crafts-type alkenylation could be a better choice to prepare such regiospecific vinyl aromatic compounds. Yamaguchi et al. reported a direct ortho alkenylation of phenols with 1-alkynes using SnCl_4_ and Bu_3_N in acetonitrile under reflux [28]. Further developments include the metal trifluoromethanesulfonate-catalyzed Friedel–Crafts alkenylation of arenes using alkynes by Tsuchimoto et al. [29] and the addition of simple arenes to arylacetylenes to afford exclusive 1,1-diarylethylenes through a C–C coupling reaction catalyzed by a combination of Cu(OTf)_2_/TMSA (TMSA = trifluoromethanesulfonic acid) [30]. The same reaction was also successfully carried out by Li and co-workers using FeCl_3_ as catalyst in a homogeneous medium [31]. Further Yadav et al. demonstrated an elegant hydroarylation of different phenols in the presence of gallium(III) chloride [32]. Nevertheless, all of these Lewis acid catalyzed Friedel–Crafts-type reactions have their own limitations. Being homogeneous catalytic systems, these methods suffer from drawbacks, such as difficulty in recovery and reusability of the catalysts and tedious work-up procedures. In addition, in most of the cases the utilization of air-sensitive chemicals also restricts these methods to be carried out under inert conditions. To overcome such limitations, heterogeneous solid catalysts have recieved much attention over the past few decades. The easy separation and possibility of reuse made their employment an attractive choice. Recently, we have successfully employed a Fe-Al-MCM-41 catalyst in a Friedel–Crafts-type hydroarylation reaction of styrenes [33]. The catalyst demonstrated high yields of products with good selectivities within a short reaction time under “open flask conditions”. On further exploration of the use of iron-based mesoporous aluminosilicate catalyst in the direct alkenylation of arenes, we have succeeded in vinylation of various phenols with different phenylacetylenes. In this paper we report a convenient method for the alkenylation of phenols with aryl-substituted alkynes under mild conditions.

## Results and Discussion

MCM-41 and Al-MCM-41 were prepared according to the procedure described in our earlier report [33]. The incorporation of iron(III) was achieved in a similar way [33]. The mesoporous patterns of MCM-41, Al-MCM-41 and Fe-Al-MCM-41 were established from the small-angle XRD patterns (see Supporting Information File 1). The BET surface area and the pore width of Fe-Al-MCM-41 were found to be 753 m^2^/g and 25.83 Å, respectively. The aluminium and iron contents of the Fe-Al-MCM-41 catalyst were estimated by AAS method and found to be 5.5 wt % and 0.80 wt %, respectively.

Initially, the Fe-Al-MCM-41-catalyzed reaction of phenylacetylene and phenol was carried out in different solvent media. The progress of the reaction was monitored by analyzing the products with the help of gas chromatography (Table 1). Amongst various solvents, such as cyclohexane, CH_3_CN, CH_3_NO_2_, CH_3_OH, CHCl_3_, 1,2-dichloroethane, and dichloromethane, cyclohexane was found to be the most suitable solvent for the reaction (Table 1, entry 7). Though a moderate yield was obtained in 1,2-dichloroethane, no conversion was observed in DCM (Table 1, entries 5–6). Comparing the reaction result in 1,2-DCE (bp 80 °C) and DCM (bp 40 °C), it seemed that temperature plays a crucial role in the activation of the substrate. Hence, further screening was done by performing the same reaction in cyclohexane at different temperatures (Table 1, entries 8 and 9). The best result was obtained at 80 °C, i.e., in cyclohexane under reflux.

**Table 1:** Fe-Al-MCM-41-catalyzed reaction of phenylacetylene and phenol^a^.

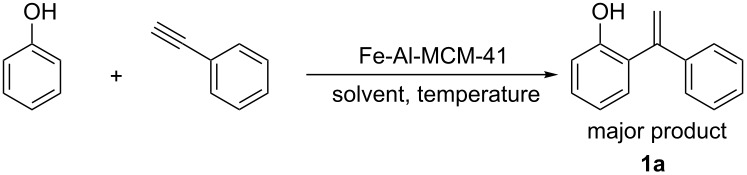					

entry	temp. [°C]	solvent	time	conversion [%]^b^	yield [%]^c^

1	80	CH_3_CN	6	0	0
2	80	CH_3_NO_2_	6	0	0
3	80	CH_3_OH	6	4	0
4	80	CHCl_3_	6	0	0
5	80	1,2-DCE	2.5	88	71
6	40	DCM	6	0	0
7	80	cyclohexane	2	99	81
8	70	cyclohexane	2.5	30	25
9	60	cyclohexane	9	1	0

^a^Reaction conditions: 1 mmol of phenylacetylene, 1.5 mmol of phenol, 65 mg of Fe-Al-MCM-41, 2 mL of solvent. ^b^GC conversion of phenylacetylene. ^c^GC yield of alkenylated product.

Since a number of pure aluminosilicates are known to catalyze C–C coupling reactions [34–42], a detailed investigation was performed with various available aluminosilicate and siliceous materials to understand the necessity of the iron center in the catalyst Fe-Al-MCM-41. NaY, a microporous aluminosilicate, did not yield any product under the specified reaction conditions (Table 2, entry 1). Sartori et al. reported an electrophilic alkenylation of aromatics with phenylacetylene over zeolite HSZ-360-Y; however, this zeolite is known to have a high number of acidic sites in its porous structure [43]. They showed that among various readily available porous aluminosilicates like HSZ-360-Y, ZSM-5-Y, K10 montmorillonite, etc., HSZ-360-Y was capable of catalyzing the electrophilic substitution process. However, a rigorous pretreatment is necessary to activate the catalyst and a moisture-free medium is essential. Furthermore, a higher temperature (110 °C) and a longer reaction time (14 h) were also required for the catalytic reaction. Sartori et. al. suggested that the external surface of HSZ-360-Y zeolite was responsible for such catalytic activity rather than the pore of the zeolite. As evidence, they reported the acidic-alumina-catalyzed reaction in which only 25% product yield was observed [43]. However, when we used acidic alumina (under our reaction conditions) at a relatively low temperature (80 °C) without any pretreatment, no conversion was observed (Table 2, entry 3). The basic and the neutral alumina also remained inactive in catalyzing the coupling reaction (Table 2, entries 4 and 5). This may be attributed to the low activation energy or the lack of moisture-free conditions. However, when iron-exchanged Al-MCM-41 was employed as catalyst the reaction proceeded smoothly to afford high yields of products within very short reaction times. The reaction was performed under “open-flask” conditions without any moisture-preventing conditions, and no pretreatment of the catalyst was required. Notably, when the alumina support “Al-MCM-41” itself was used as catalyst, the conversion recorded was only 2% (Table 2, entry 7). The aluminium-free pure mesoporous silica, MCM-41, displayed no catalytic activity, as expected (Table 2, entry 8). This clearly indicates that the presence of iron in the catalyst Fe-Al-MCM-41 is directly related to its superior performance in the catalytic reaction. Iron probably has some role in lowering the activation energy of the reaction in synergy with the aluminium moiety present in Al-MCM-41. The short reaction time is probably due to the mesoporous structure of Fe-Al-MCM-41 (pore width = 25.83 Å), which provides a higher surface area to facilitate the reaction. It is well established that the site-isolation of active centers enhances the catalytic efficacy of the materials [44–47]. Owing to the large surface area of catalyst Fe-Al-MCM-41, iron centers are well dispersed in the mesoporous matrix. Thus, the catalyst attained the desired site-isolation of active centers for enhanced activity in catalytic reaction. In fact, in order to investigate the role of mesoporous structure, we further performed the reaction with “degraded-Fe-Al-MCM-41” of which the mesoporous integrity was destroyed by proper treatment [48]. In that case, a long reaction time was observed (Table 2, entry 9), as in the case of HSZ-360-Y.

**Table 2:** Reactions of phenylacetylene with phenol with different catalysts^a^.

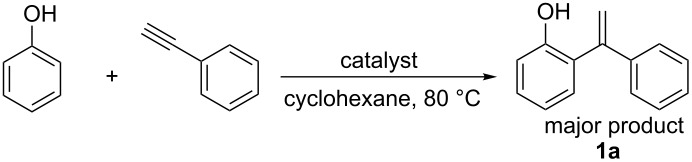				

entry	catalyst	time (h)	conversion [%]^b^	yield [%]^c^

1	NaY	6	0	0
2^d^	HSZ-360-Y	14	55	52
3	acidic alumina	6	3	0
4	basic alumina	6	0	0
5	neutral alumina	6	0	0
6	Fe-Al-MCM-41	2	99	81
7	Al-MCM-41	6	2	0
8	MCM-41	6	0	0
9^e^	Fe-Al-MCM-41 (degraded)	12	48	45
10^f^	GaCl_3_	6	–	75
11^g^	FeCl_3_·6H_2_O (10 mol %)	12	0	0

^a^Reaction conditions: 1 mmol of phenylacetylene, 1.5 mmol of phenol, 65 mg of catalyst, 2 mL of cyclohexane, 80 °C. ^b^GC conversion of phenylacetylene. ^c^GC yield of arylated products. ^d^Ref [43]. ^e^See Experimental for details. ^f^Ref [32]. ^g^Catalyst: 2.32 mg of FeCl_3_·6H_2_O (0.008 mmol).

On the basis of the optimized reaction conditions, the scope of this Fe-Al-MCM-41 catalyzed C–C coupling reaction was further investigated. Various substituted phenols were used for the alkenylation by phenylacetylene. The reaction preceded smoothly both in the case of electron-donating and -withdrawing groups at the phenol. While *p*-Br-, *p*-Me- and *p*-OMe-phenol took about 1–2 hours for a good conversion, *p*-Cl-phenol took slightly longer (viz. 2.5 hours) (Table 3, entries 2–5). In all cases a mono-alkenylated product at the ortho position was observed in high yield. Moreover, the catalyst showed regioselectivity in producing the 1,1-diarylalkene product. For alkynes, *p*-Me-substituted phenylacetylene was further used to explore the general acceptability of this reaction. In its reaction with different substituted phenols a lowering of the reaction time was observed (Table 3, entries 6–9). Thus, Fe-Al-MCM-41 exhibited its efficiency in catalyzing the alkenylation of different phenols by various substituted/nonsubstituted phenylacetylenes to furnish the corresponding 1,1-diarylalkenes in high conversion and selectivity within a short reaction time.

**Table 3:** Reactions of arenes with phenylalkynes^a^_._

entry	arene	product^b^	time [h]	conversion [%]^c^	yield [%]^d^

1	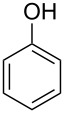	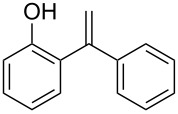 **1a**	2	99	81
2	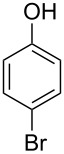	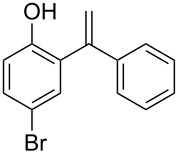 **1b**	1	100	83
3	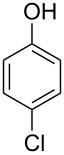	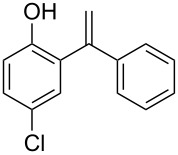 **1c**	2.5	93	75
4	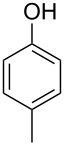	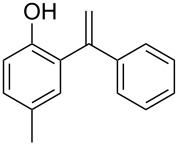 **1d**	1.5	99	87
5	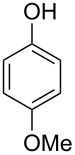	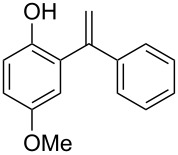 **1e**	2	99	89
6	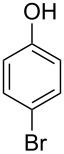	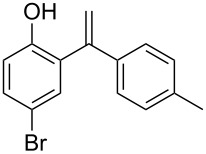 **2a**	0.75	100	86
7	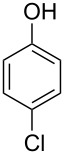	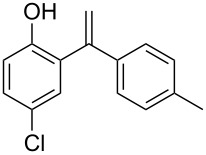 **2b**	2	97	81
8	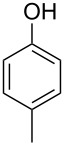	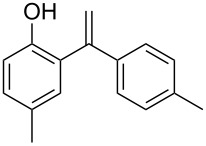 **2c**	1	100	87
9	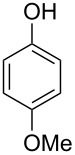	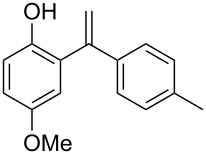 **2d**	1.5	100	89

^a^Reaction conditions: 1 mmol of arylalkyne, 1.5 mmol of arene, 65 mg of Fe-Al-MCM-41, 2 mL of solvent, 80 °C. ^b^main product. ^c^GC conversion of phenylalkyne. ^d^GC yield of alkenylated product.

Furthermore, to verify whether the coupling reaction was truly catalyzed by solid Fe-Al-MCM-41 or by the leached iron species from the solid support, a “hot filtration” experiment was undertaken. A reaction between phenol and phenylacetylene was performed until 22% conversion was observed (monitored by GC analysis) and then the catalyst was separated by simple centrifugation under the hot conditions. No conversion in the “catalyst-free centrifugate” was observed upon further heating for 12 hours.

Another most advantageous aspect of this type of solid-support catalyst is their scope for recycling. The reaction between phenol and phenylacetylene under optimized conditions was chosen to evaluate the recyclability of Fe-Al-MCM-41. The catalyst was separated after the completion of the reaction by simple centrifugation. This recovered catalyst was washed with cyclohexane, then methanol followed by dichloromethane, and dried at 80 °C for a few hours prior to its next use. The conversions of successive cycles are given in Table 4.

**Table 4:** Recycling of Fe-Al-MCM-41 catalyst for the reaction of phenol with phenylacetylene.

	cycle			

	1	2	3	4

conversion (%)^a^	99	97	98	96
time (h)	2	2	2	2
yield (%)^b^	81	80	80	78

^a^GC conversion of phenylacetylene. ^b^GC yield of arylated products.

## Conclusion

In summary, Fe-Al-MCM-41 has been proven to be an efficient catalyst for the alkenylation of phenols with aryl-substituted alkynes. Various substituted arylalkynes and phenols afforded the 1,1-diarylethylenes under mild reaction conditions. The reaction did not require any further addition of acids or special reagents such as additives, etc. The catalytic reactions can be carried out under “open-flask conditions”. Furthermore, the catalyst can be easily recovered from the reaction mixture by simple centrifugation and can be reused several times without any special treatment other than washing.

## Experimental

**General procedure for catalytic reactions:** Phenylacetylene (1.0 mmol) was added to a mixture of phenol (1.5 mmol) and Fe-Al-MCM-41 (0.065 g) in 2 mL of cyclohexane. The mixture was stirred at 80 °C in an oil bath. To study the progress of the reaction, the products were collected at different time intervals and identified and quantified by gas chromatography. After completion of the reaction, the solution was cooled down and the catalyst was removed by centrifugation. The resulting crude mixture was gently evaporated under vacuum and purified by flash column chromatography on silica gel 230–400 by using an appropriate solvent.

**2-(1-phenylvinyl)phenol** (**1a**) [43]: ^1^H NMR (300 MHz, CDCl_3_) δ 7.40–7.32 (m, 5H, C*H*), 7.30–7.13 (m, 3H, C*H*), 6.97–6.94 (m, 2H, C*H*), 5.88 (d, *J*(H,H) = 0.6 Hz, 1H, CH*H*), 5.43 (s, 1H, C*H*H), 5.18 (s, 1H, O*H*).

**Synthesis of the catalyst (Fe-Al-MCM-41):** As described in our previous work [33], iron was incorporated into the mesoporous aluminosilicate (Al-MCM-41) by the ion-exchange method. A 0.5 g amount of Al-MCM-41 was added to 100 mL 0.001 M methanolic solution of FeCl_3_·6H_2_O, and the mixture was stirred vigorously for 12 h. The resultant solid was then filtered. To remove the excess FeCl_3_, the isolated solid was washed by Soxhlet extraction using methanol. The resulting solid was dried in an oven at 80 °C and characterized. The catalyst was then used directly in reactions without any further activation.

**Disintegration of mesoporous structure:** The mesoporous structure is known to disintegrate during boiling with water, due to silicate hydrolysis [48]. The disintegration of the mesoporous structure was achieved by boiling Fe-Al-MCM-41 with millipore water. The liquid-to-sample ratio was fixed at 1 Lg^−1^. After 12 h of heating, the sample was filtered and dried in an oven for 2 h at 398 K. The XRD patterns of the dried sample revealed that the mesoporous integrity was totally lost. The catalytic reaction was performed with this “degraded-Fe-Al-MCM-41” (Table 2, entry 9).

## Supporting Information

File 1 Experimental procedures with characterization data for all compounds.
