# Supplementary material for: Iron-containing mesoporous aluminosilicate catalyzed direct alkenylation of phenols: Facile synthesis of 1,1-diarylalkenes
Source: Beilstein J Org Chem. 2013 Jan 9;9:49–55. doi: 10.3762/bjoc.9.6 (PMC3566757; doi:10.3762/bjoc.9.6)
Supplement: File 1 — Experimental procedures with characterization data for all compounds. [file Beilstein_J_Org_Chem-09-49-s001.pdf]

Supporting Information

for

Iron-containing mesoporous aluminosilicate catalyzed direct alkenylation of phenols: Facile synthesis of 1,1-diarylalkenes

Satyajit Haldar and Subratanath Koner*

Address: Department of Chemistry, Jadavpur University, Kolkata 700032, India.

Email: Subratanath Koner - snkoner@chemistry.jdvu.ac.in

* Corresponding author

Experimental procedures with characterization data for all compounds

Table of Contents

Page S2: General Information.

Pages S2–S3: Synthesis of Al-MCM-41, Fe-Al-MCM-41, degradation of mesoporous structure and their XRD study.

Page S4: Nitrogen-sorption studies of Fe-Al-MCM-41.

Pages S5–S8: General experimental procedure of organic reaction, spectral data.

Pages S9–S23: ^{1}H NMR and ^{13}C NMR charts for all compounds.

General information

All materials were purchased from commercial suppliers and used without further purification. $FeCl_3·6H_2O$ was purchased from Aldrich (iron(III) chloride hexahydrate, reagent grade, ≥98%, purified lumps). The powder X-ray diffraction (XRD) patterns of the samples were recorded with an X-ray diffractometer using Cu K_α radiation. Prior to N_2-sorption experiments samples were outgassed at 120 °C. All reactions were carried out in air, without any special precautions. Flash column chromatography was performed over silica gel (mesh 230–400) and hexane/ethyl acetate combination was used as the eluent. ^{1}H NMR and ^{13}C NMR spectra were recorded at ambient temperature in $CDCl_3$ with tetramethylsilane as internal standard. The chemical shifts (δ) and coupling constants (*J*) were expressed in ppm and Hz, respectively. Gas chromatography was performed to identify and quantify the products of the reaction. Infra-red spectra were recorded using a neat sample. HRMS measurements were performed on a mass spectrometer by electron-spray-ionization method.

Synthesis of Al-MCM-41, Fe-Al-MCM-41, degraded "Fe-Al-MCM-41" and their XRD study:

Synthesis of Al-MCM-41 [1]: As described earlier [1], for the synthesis of the Al-MCM-41 (Si/Al = 16) material, 22.3 mL (1 mol) of tetraethylorthosilicate was mixed with 0.68 g (0.033 mol) of aluminium isopropoxide (dissolved in 5 mL of distilled water). The mixture was stirred for 30 minutes and tetraethylammonium hydroxide solution (10% water) was added under continued stirring for another 30 minutes until gel formation (pH = 11). After that, 7.2 g (0.2 mol) of cetyltrimethylammonium bromide was added dropwise (30 mL/h) so that the gel changed into a suspension. After further stirring for 1 h the resulting synthesis gel of composition $1SiO_2:0.033Al_2O_3:0.2CTMABr:100H_2O$ was transferred into a Teflon-line steel autoclave and heated to 150 °C for 48 h. After cooling to room temperature, the material was recovered by filtration, washed with deionized water and ethanol, dried in air at 100 °C for 1 h and finally calcined under a flow of air at 540 °C for 6 h.

Synthesis of Fe-Al-MCM-41 [1]: As described earlier [1], iron was incorporated into the mesoporous aluminosilicate (Al-MCM-41) using a methanolic $FeCl_3$ solution. 0.5 g of Al-MCM-41 was added to 100 mL 0.001 M methanolic solution of $FeCl_3 \cdot 6H_2O$ and stirred vigorously for 12 h. The resultant solid was then filtered. To remove the excess $FeCl_3$, it was washed with Soxhlet exraction using methanol. The resulting solid was dried in an oven at 80 °C and characterized by SAX and nitrogen sorption analysis.

Disintegration of mesoporous structure: The disintegration of mesoporous structure was done by boiling Fe-Al-MCM-41 with millipore water. The liquid-to-sample ratio was fixed as 1 Lg^{-1}. After 12 h of heating, the sample was filtered and dried in an oven for 2 h at 398 K. The XRD pattern of the dried sample was analyzed.

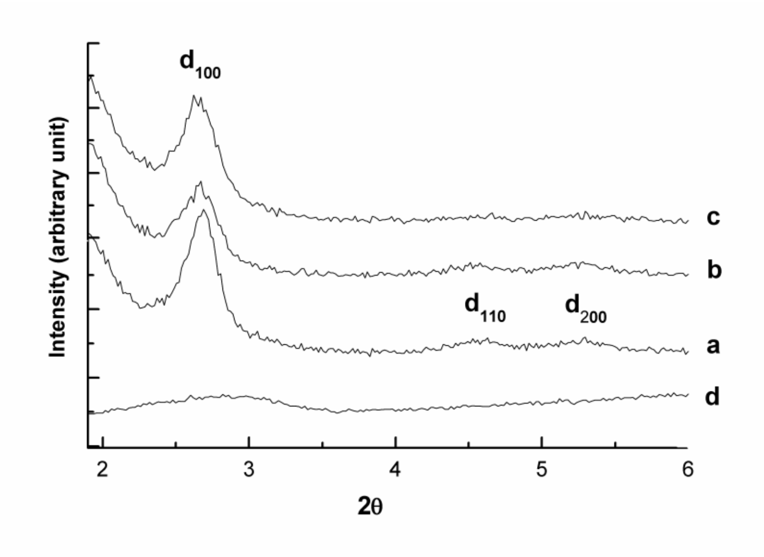

Figure S1: Small-angle X-ray (SAX) diffraction patterns of (a) calcined Al-MCM-41, (b) Fe-Al-MCM-41, (c) recovered Fe-Al-MCM-41(d) “degraded-Fe-Al-MCM-41”.

Nitrogen-sorption studies of Fe-Al-MCM-41

The nitrogen-sorption experiments showed that the MCM-41 has the BET surface area (A_{sBET}) of 753 m^2g^{-1}. The average pore diameter is calculated to be 25.83 Å for Fe-Al-MCM-41.

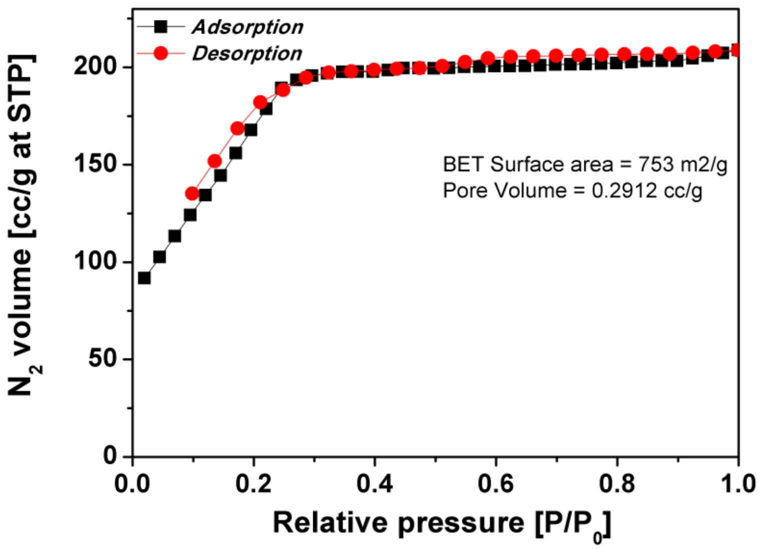

Figure S2: N_2 adsorption/desorption isotherms of Fe-Al-MCM-41. Adsorption points are marked by black filled squares and desorption ones by red filled circles.

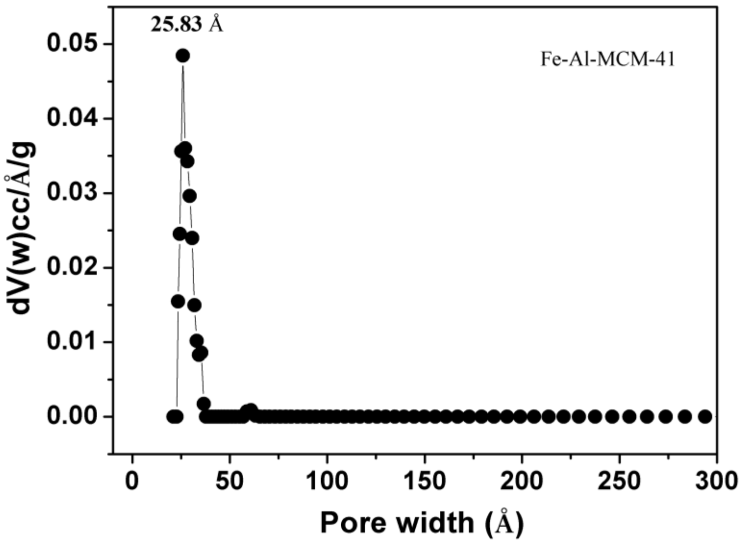

Figure S3: Pore size distribution curve of Fe-Al-MCM-41

General procedure of organic reaction and spectral data: Phenylacetylene (1.0 mmol) was added to a mixture of phenol (1.5 mmol) and Fe-Al-MCM-41 (0.065 g) in 2 mL of cyclohexane. The mixture was stirred at 80 °C in an oil bath. To study the progress of the reaction the products were collected at different time intervals and identified and quantified by gas chromatography. After completion of the reaction, the solution was cooled down and the catalyst was removed by centrifugation. The resulting crude mixture was gently evaporated under vacuum and purified by flash column chromatography on silica gel 230–400 using an appropriate solvent.

2-(1-Phenylvinyl)phenol (1a) [2]:

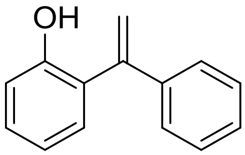

^{1}H NMR (300 MHz, $CDCl_3$) δ 7.40–7.32 (m, 5H, C*H*), 7.30–7.13 (m, 3H, C*H*), 6.97–6.94 (m, 2H, C*H*), 5.88 (d, *J*(H,H) = 0.6 Hz,1H, CH*H*), 5.43 (s, 1H, C*H*H), 5.18 (s, 1H; O*H*).

4-Bromo-2-(1-phenylvinyl)phenol (1b):

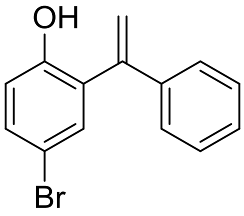

^{1}H NMR (300 MHz, $CDCl_3$) δ 7.37–7.34 (m, 6H, C*H*), 7.29 (d, *J*(H,H) = 2.4 Hz, 1H, C*H*), 6.84 (d, *J*(H,H) = 8.6 Hz, 1H, C*H*), 5.88 (d, *J*(H,H) = 0.6 Hz, 1H, CH*H*), 5.43 (s, 1H, C*H*H), 5.15 (s, 1H, O*H*); ^{13}C NMR (75 MHz, $CDCl_3$) δ 152.3, 144.2, 138.6, 132.7, 132.2, 129.6, 128.9, 128.8, 126.9, 117.7, 117.5, 112.5; IR (neat, liquid): ν_{max} = 3515, 3081, 3057, 3028, 1599, 1477, 1404, 1331, 1268, 1193, 912, 818, 781, 706 cm^{-1}; HRMS (ESI–TOF): $[M + Na]^+$, calcd. for $C_{14}H_{11}BrONa$: 296.9891, found 296.9887.

4-Chloro-2-(1-phenylvinyl)phenol (1c) [3]:

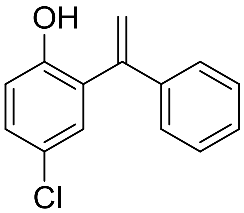

^{1}H NMR (300 MHz, $CDCl_3$) δ 7.36 (bs, 5H, C*H*), 7.21 (dd, *J*(H,H) = 2.6 Hz, *J*(H,H) = 8.6 Hz, 1H, C*H*), 7.13 (d, *J*(H,H) = 2.6 Hz, 1H, C*H*), 6.88 (d, *J*(H,H) = 8.7 Hz, 1H, C*H*), 5.88 (d, *J*(H,H) = 1 Hz, 1H, CHH), 5.43 (d, *J*(H,H) = 1 Hz, 1H, CHH), 5.10 (s, 1H, OH); ^{13}C NMR (75 MHz, $CDCl_3$) δ 151.8, 144.4, 138.7, 129.9, 129.3, 129.1, 128.9, 128.8, 127.0, 125.3, 117.5, 117.3; IR (neat, liquid): ν_{max} = 3521, 3060, 3029, 2362, 1600, 1480, 1408, 1331, 1269, 1194, 1114, 915, 820, 781cm^{-1}; HRMS (ESI–TOF): [M + Na]$^+$, calcd. for $C_{14}H_{11}ClONa$: 253.0396, found 253.0394.

4-Methyl-2-(1-phenylvinyl)phenol (1d) [4]:

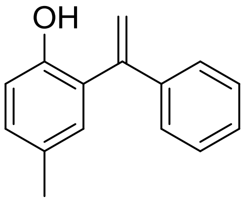

^{1}H NMR (300 MHz, $CDCl_3$) δ 7.40–7.33 (m, 5H, C*H*), 7.08–6.84 (m, 3H, C*H*), 5.85 (d, *J*(H,H) = 1.2 Hz, 1H, CHH), 5.41 (d, *J*(H,H) = 1.0 Hz,1H, CHH), 5.02 (s, 1H, O*H*), 2.28 (s, 3H, CH_3).

4-Methoxy-2-(1-phenylvinyl)phenol (1e) [4]:

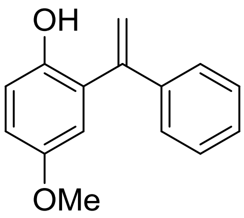

^{1}H NMR (300 MHz, $CDCl_3$) δ 7.39–7.33 (m, 5H, C*H*), 6.90–6.84 (m, 2H, C*H*), 6.70 (d, *J*(H,H) = 2.8 Hz,1H, CH), 5.87 (s, 1H, CHH), 5.43 (s, 1H, CHH), 4.81 (s, 1H, OH), 3.75 (s, 3H, OCH_3).

4-Bromo-2-(1-*p*-tolylvinyl)phenol (2a) [5]:

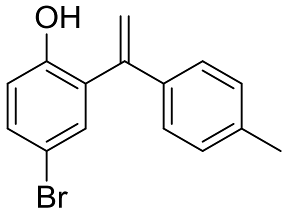

^{1}H NMR (300 MHz, $CDCl_3$) δ 7.38–7.16 (m, 6H, C*H*), 6.84 (d, *J*(H,H) = 8.6 Hz, 1H, C*H*), 5.84 (s, 1H, CHH), 5.37 (s, 1H, CHH), 5.18 (bs, 1H, O*H*), 2.38 (s, 3H, CH_3); ^{13}C NMR (75 MHz, $CDCl_3$) δ 152.4, 144.0, 139.0, 135.7, 132.7, 132.2, 129.8, 129.6, 126.9, 117.7, 116.6, 112.5, 21.2; IR (neat, liquid): ν_{max} = 3027, 2921, 2865, 2361, 1606, 1479, 1402, 1332, 1266, 1195, 910, 826, 733 cm^{-1}; HRMS (ESI–TOF): $[M + Na]^+$, calcd. for $C_{15}H_{13}BrONa$: 311.0047, found 311.0042.

4-Chloro-2-(1-*p*-tolylvinyl)phenol (2b):

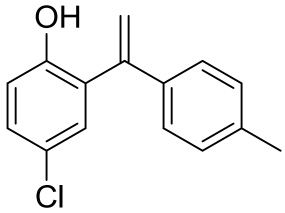

^{1}H NMR (300 MHz, $CDCl_3$) δ 7.27–7.14 (m, 6H, C*H*), 6.88 (d, *J*(H,H) = 8.7 Hz, 1H, C*H*), 5.84 (s, 1H, CHH), 5.37 (s, 1H, CHH), 5.11 (bs, 1H, O*H*), 2.37 (s, 3H, CH_3); ^{13}C NMR (75 MHz, $CDCl_3$) δ 151.8, 144.1, 139.0, 135.7, 129.9, 129.6, 129.2, 128.5, 126.9, 125.2, 117.2, 116.6, 21.2; IR (neat, liquid): ν_{max} = 3517, 3028, 2922, 2865, 1667, 1605, 1477, 1410, 1333, 1268, 1195, 1114, 1019, 910, 826, 733 cm^{-1}; HRMS (ESI–TOF): $[M + Na]^+$, calcd. for $C_{15}H_{13}ClONa$: 267.0553, found 267.0556.

4-Methyl-2-(1-*p*-tolylvinyl)phenol (2c):

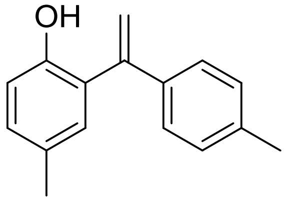

^{1}H NMR (300 MHz, $CDCl_3$) δ 7.32–7.29 (m, 2H, C*H*), 7.18 (d, *J*(H,H) = 7.9 Hz, 2H, C*H*), 7.10–7.07 (m, 1H, C*H*), 6.99 (d, *J*(H,H) = 0.7 Hz, 1H, C*H*), 6.89–6.86 (m, 1H, C*H*), 5.83 (d, *J*(H,H) = 1.2 Hz, 1H, CHH), 5.38 (d, *J*(H,H) = 1.2 Hz, 1H, CHH), 5.07 (s, 1H, O*H*), 2.39 (s, 3H, CH_3), 2.31 (s, 3H, CH_3): ^{13}C NMR (75 MHz, $CDCl_3$) δ 151.0, 145.3, 138.5, 136.7, 130.7, 129.9, 129.5, 129.4, 127.5, 127.0, 115.6, 21.2, 20.4; IR (neat, liquid): ν_{max} = 3528, 3025, 2921, 2863, 2361, 1608, 1495, 1336, 1277, 1218, 1188, 1019, 907, 827, 734 cm^{-1}; HRMS (ESI–TOF): $[M + Na]^+$, calcd. for $C_{16}H_{16}ONa$: 247.1099, found 247.1099.

4-Methoxy-2-(1-*p*-tolylvinyl)phenol (2d):

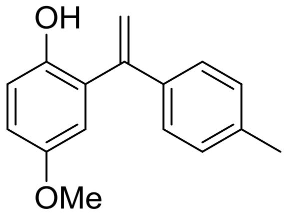

^{1}H NMR (300 MHz, $CDCl_3$) δ 7.30–7.26 (m, 2H, C*H*), 7.17–7.14 (m, 2H, C*H*), 6.90–6.84 (m, 2H, C*H*), 6.71 (d, *J*(H,H) = 2.7 Hz, 1H, C*H*), 5.82 (s, 1H, CHH), 5.37 (s, 1H, CHH), 4.83 (s, 1H, O*H*), 3.76 (s, 3H, OCH_3), 2.36 (s, 3H, CH_3); ^{13}C NMR (75 MHz, $CDCl_3$) δ 153.4, 147.2, 145.2, 138.6, 136.3, 129.5, 128.4, 126.9, 116.6, 115.9, 115.4, 115.1, 55.8, 21.2; IR (neat, liquid): ν_{max} = 3528, 2999, 2945, 2833, 1672, 1669, 1611, 1605, 1499, 1217, 1038, 828, 732 cm^{-1}; HRMS (ESI–TOF): $[M + Na]^+$, calcd. for $C_{16}H_{16}O_2Na$: 263.1048, found 263.1045.

References

1. Haldar, S.; Koner, S. *J. Org. Chem.* **2010**, *75*, 6005-6008.
2. Yadav, J. S.; Reddy, B. V. S.; Sengupta, S.; Biswas, S. K. *Synthesis* **2009**, *8*, 1301-1304
3. a) Casiraghi, G.; Casnati, G.; Puglia, G.; Sartori, G.; Terenghi, G. *Synthesis* **1977,** *2*, 122-124.
b) Moure, M. J.; SanMartin, R.; Dominguez, E. *Angew. Chem. Int. Ed.* **2012**, *51*, 3220-3224.
4. Wang, X.; Guram, A.; Caille, S.; Hu, J.; Preston, J P.; Ronk, M.; Walker, S. *Org. Lett.* **2011**, *13*, 1881-1883.
5. Song, T. K.; Teng, M.; Chandraratna, R. A. Trisubstituted phenyl derivatives having retinoid agonist, antagonist or inverse agonist type biological activity. PCT Int. Appl. WO 98/47854 A1

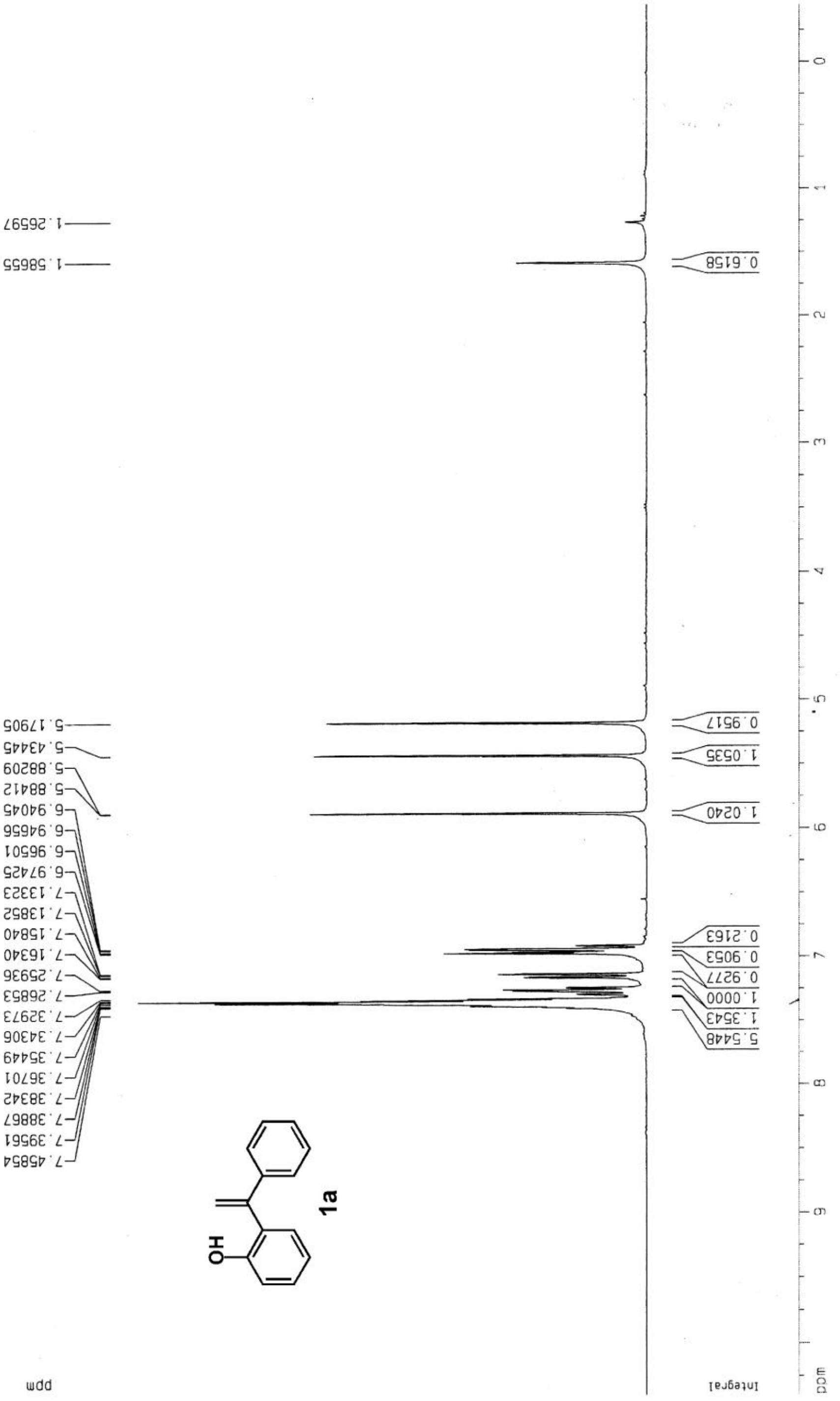

^{1}H NMR of 2-(1-phenylvinyl)phenol (**1a**)

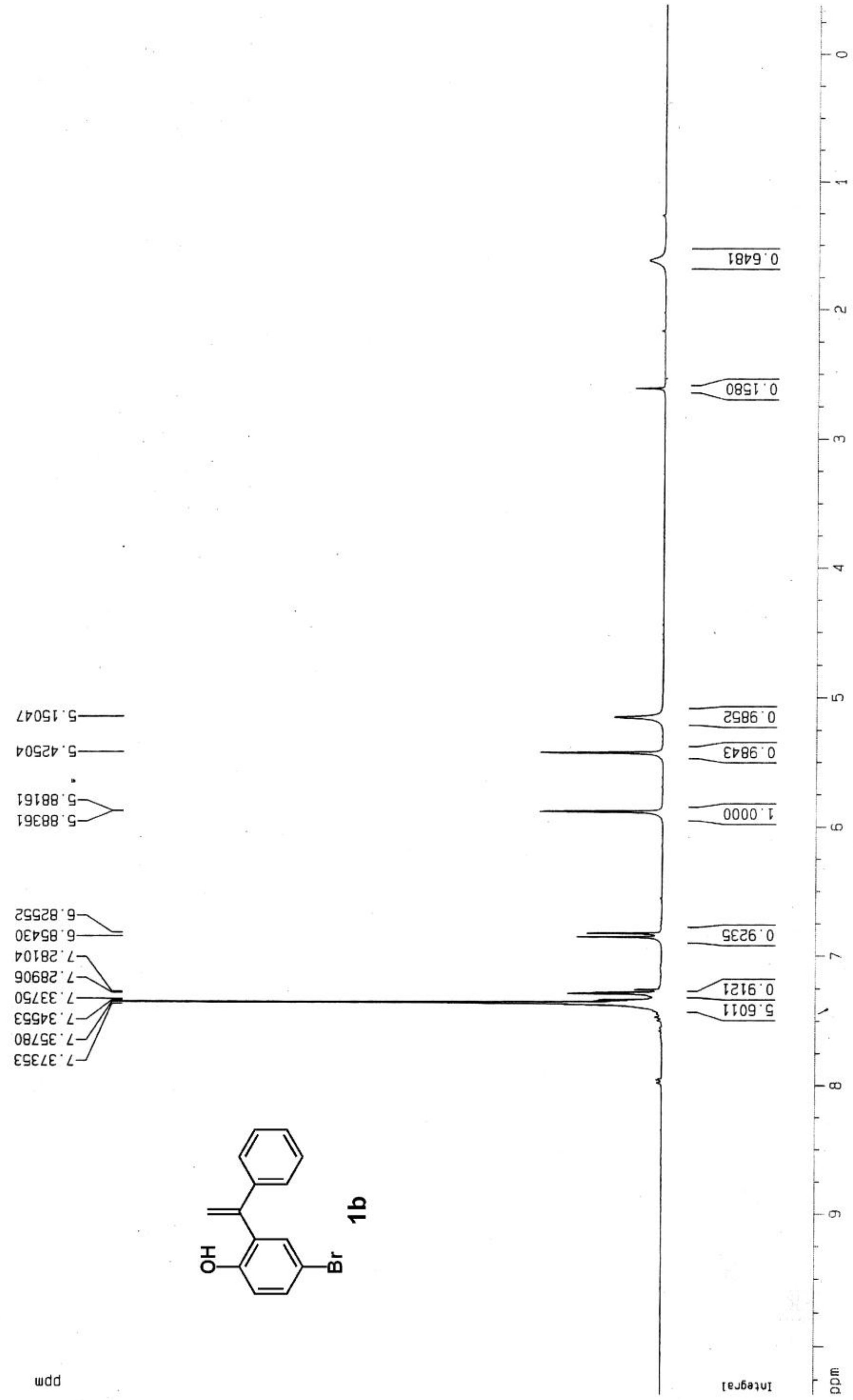

^{1}H NMR of 4-bromo-2-(1-phenylvinyl)phenol (**1b**)

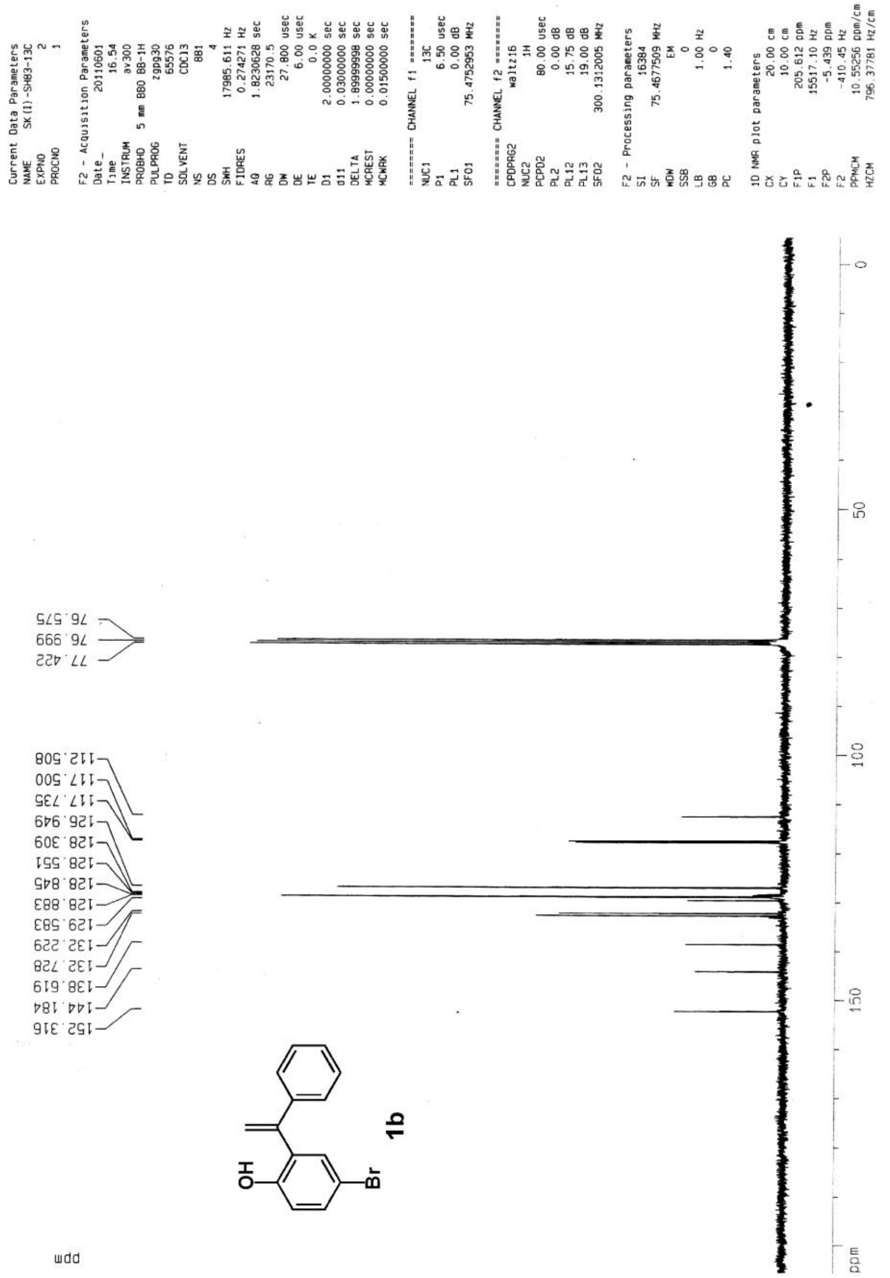

^{13}C NMR of 4-bromo-2-(1-phenylvinyl)phenol (**1b**)

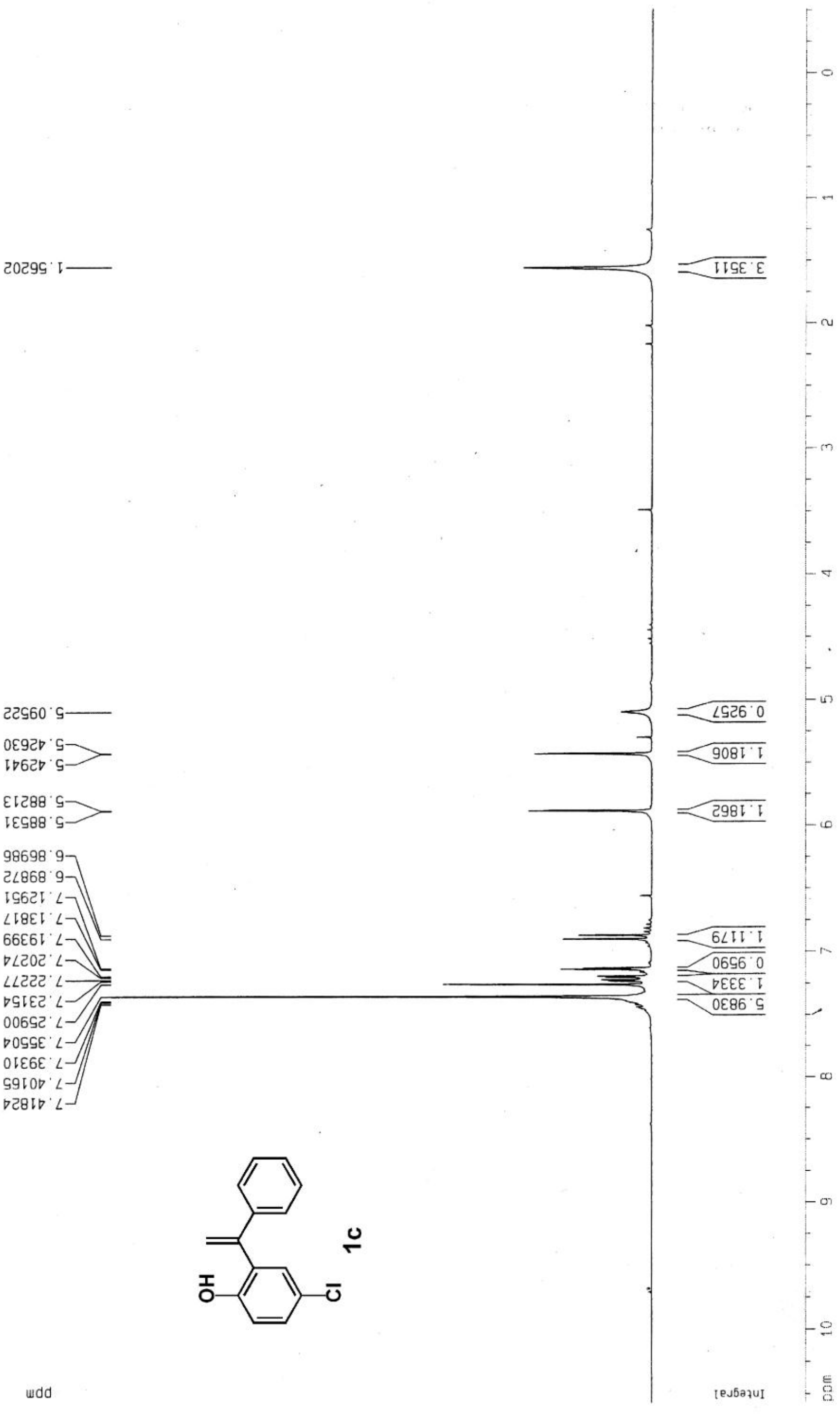

^{1}H NMR of 4-chloro-2-(1-phenylvinyl)phenol (**1c**)

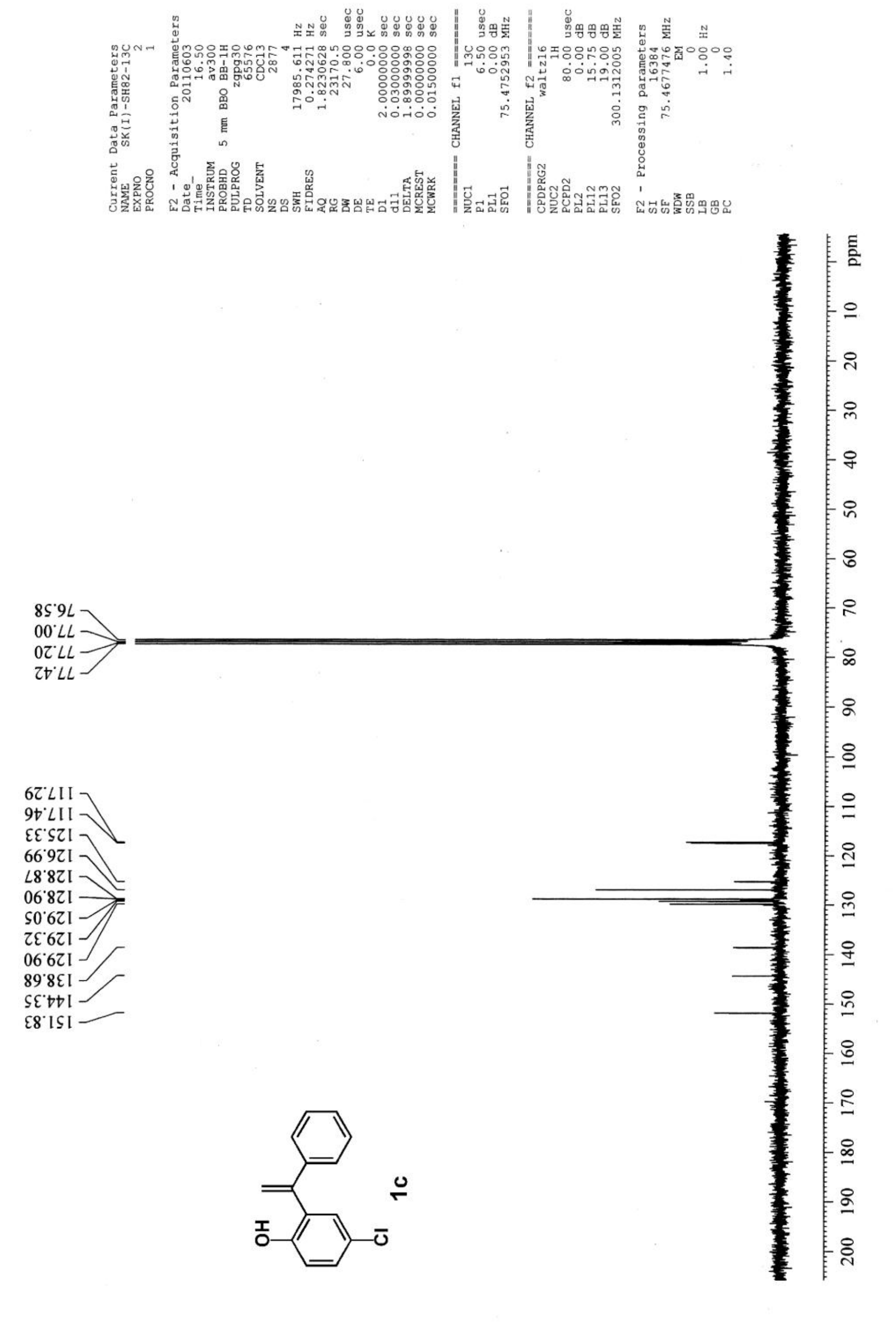

^{13}C NMR of 4-chloro-2-(1-phenylvinyl)phenol (**1c**)

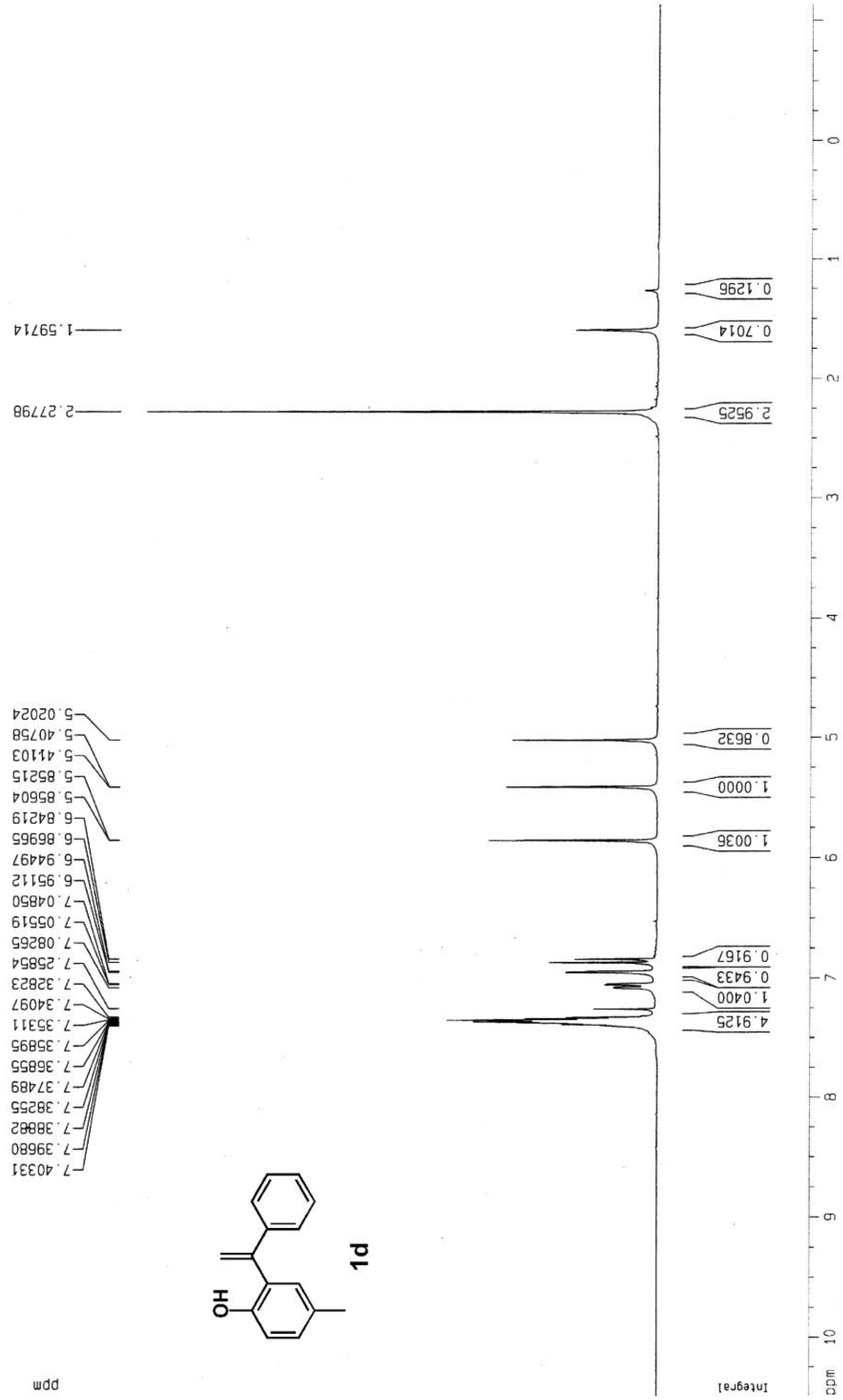

^{1}H NMR of 4-methyl-2-(1-phenylvinyl)phenol (**1d**)

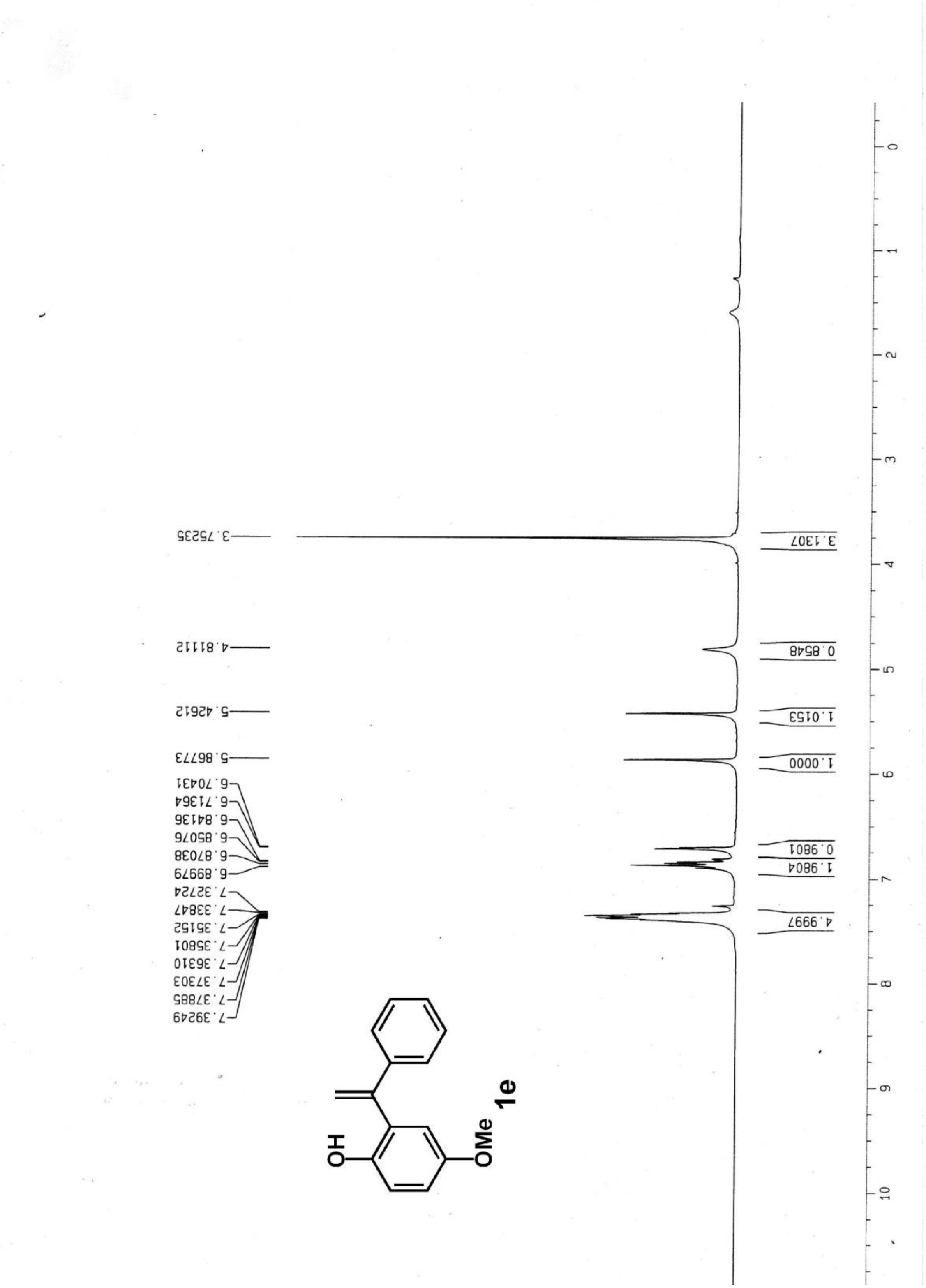

^{1}H NMR of 4-methoxy-2-(1-phenylvinyl)phenol (**1e**)

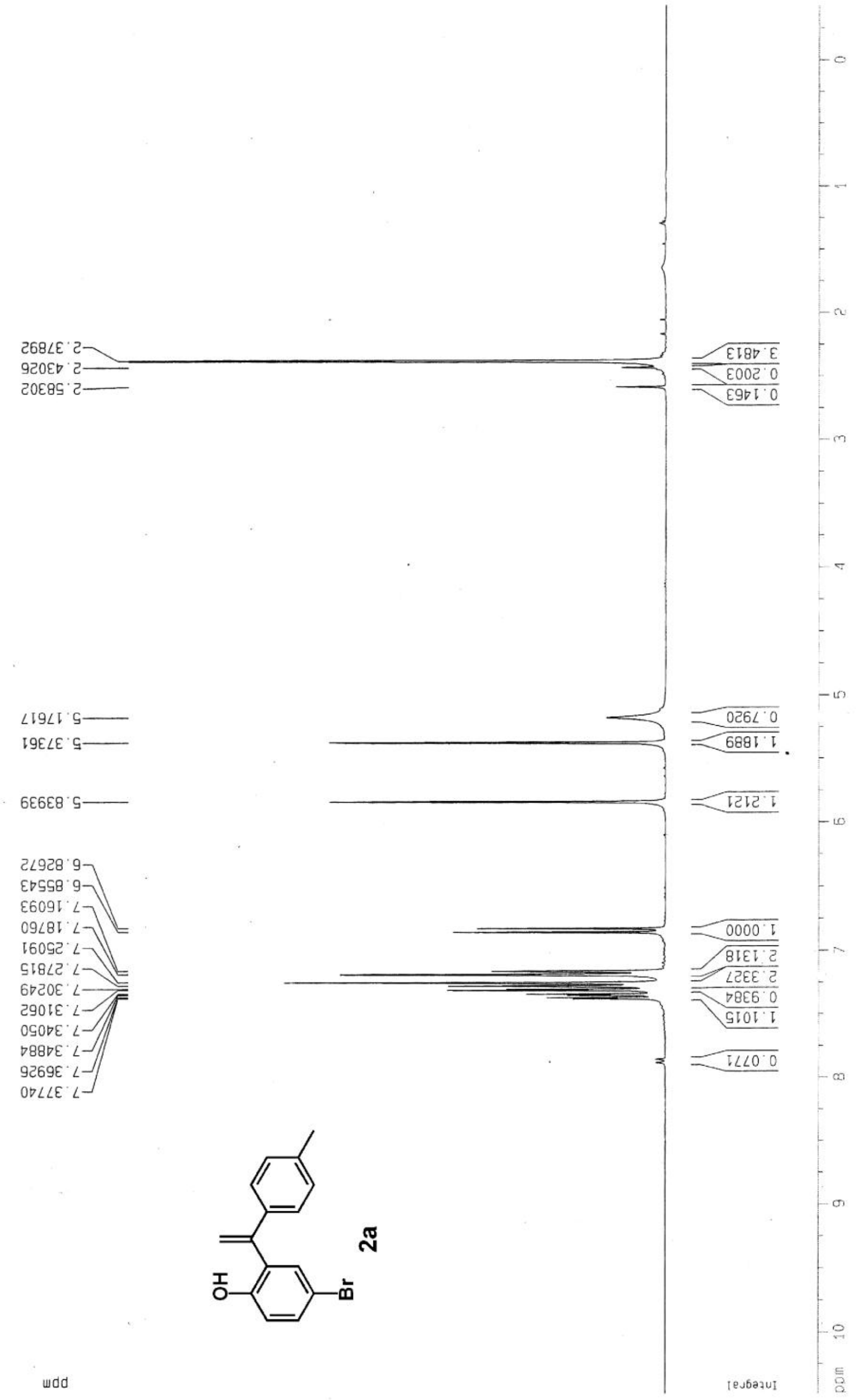

^{1}H NMR of 4-bromo-2-(1-*p*-tolylvinyl)phenol (**2a**)

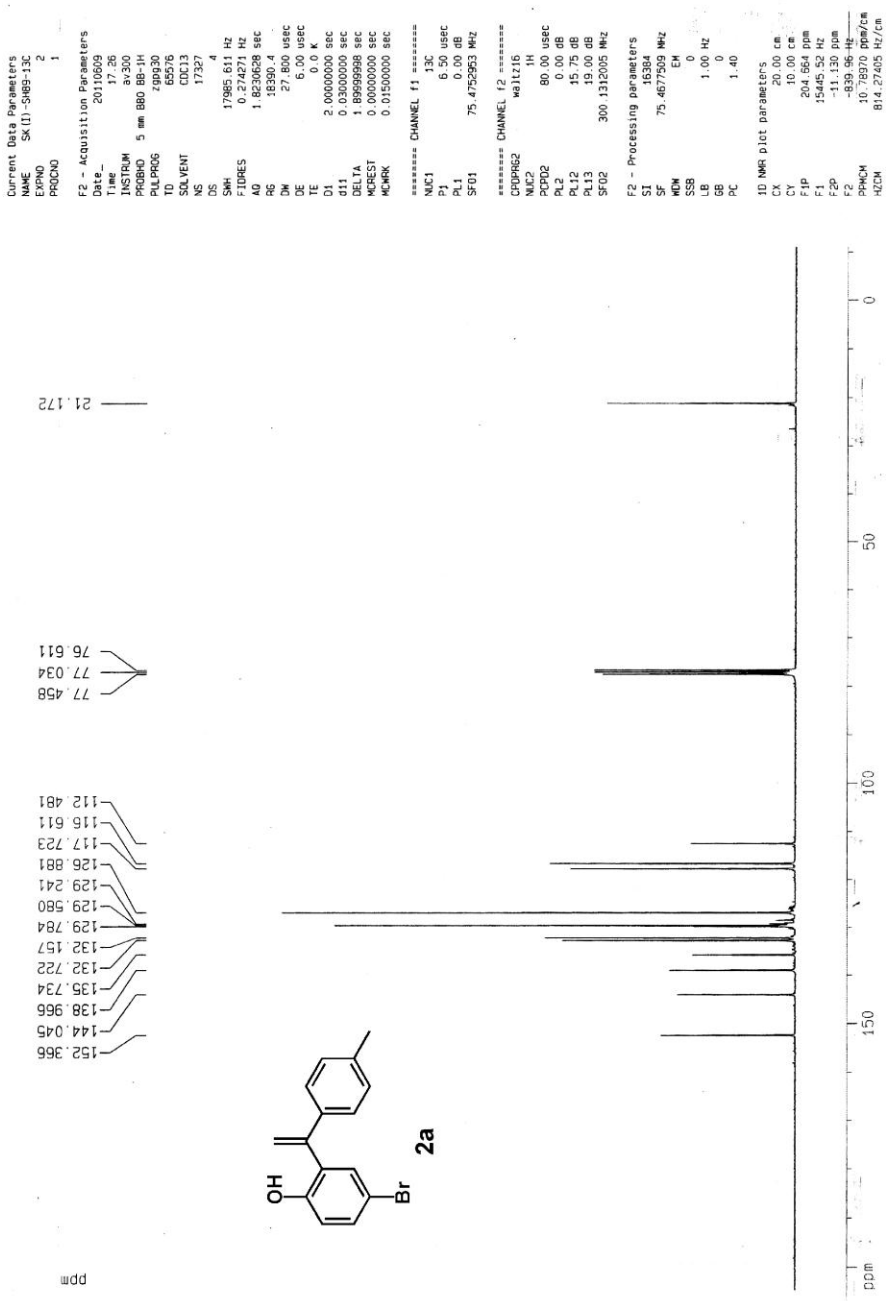

^{13}C NMR of 4-bromo-2-(1-*p*-tolylvinyl)phenol (**2a**)

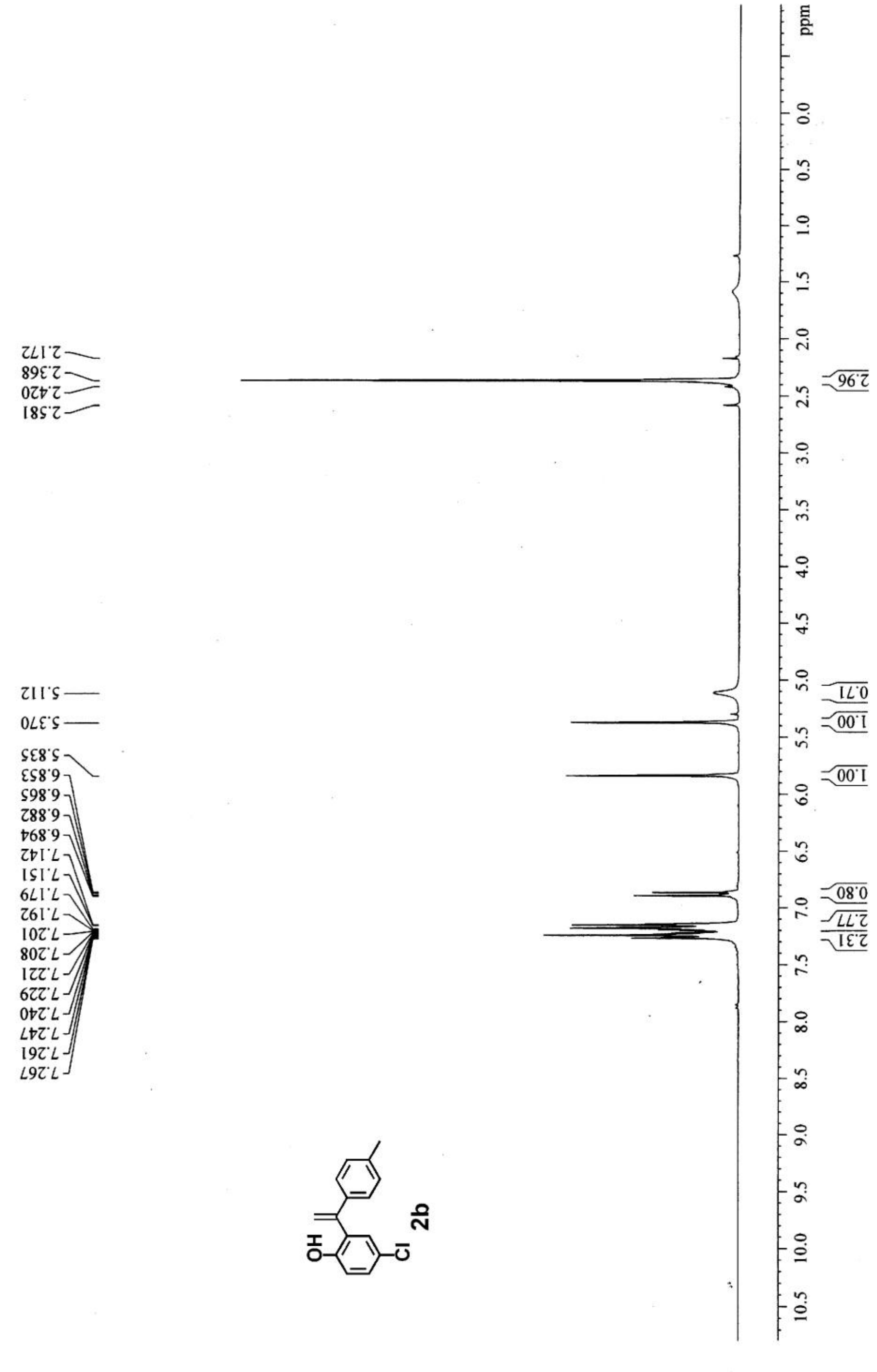

^{1}H NMR of 4-chloro-2-(1-*p*-tolylvinyl)phenol (**2b**)

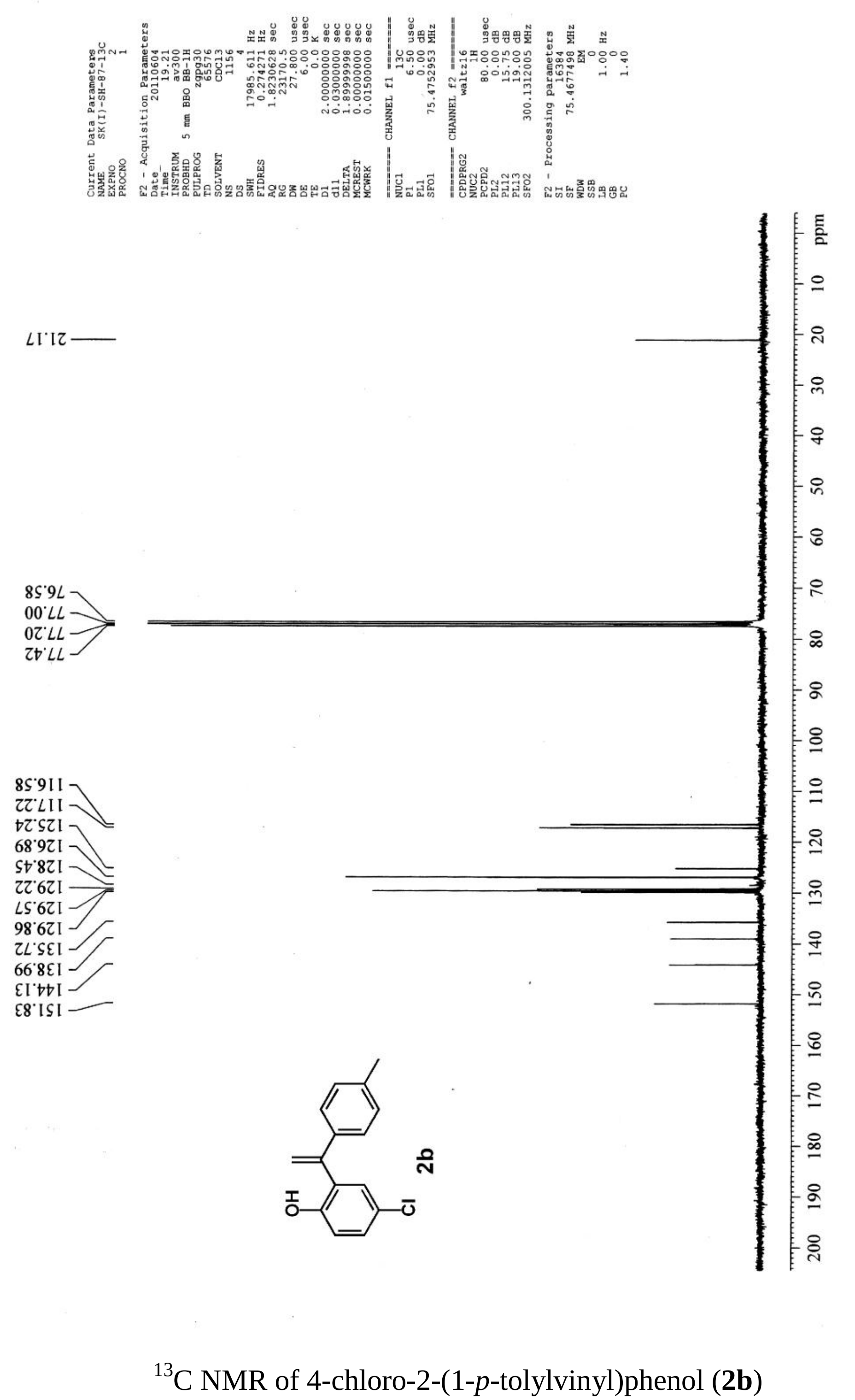

^{13}C NMR of 4-chloro-2-(1-*p*-tolylvinyl)phenol (**2b**)

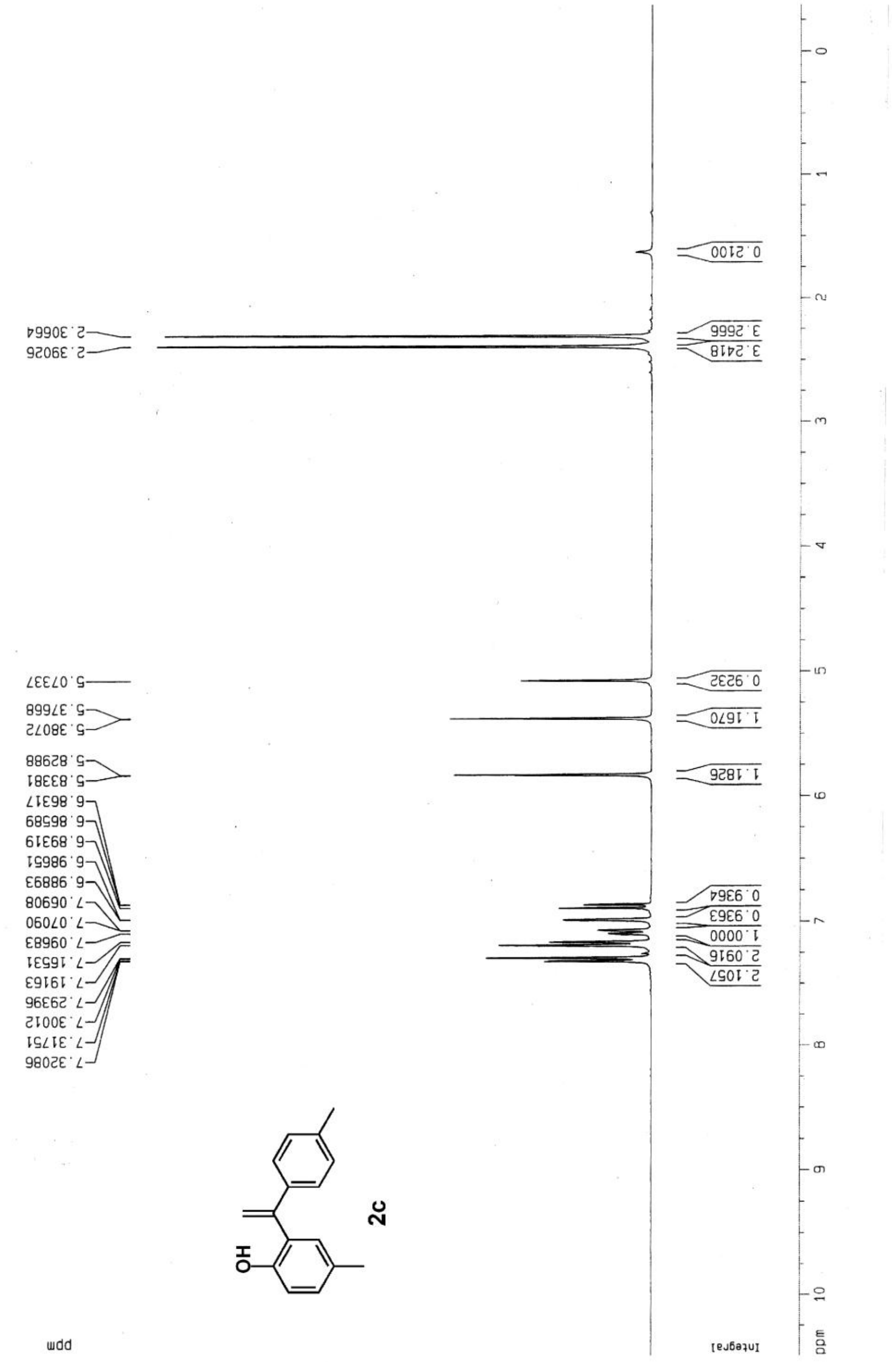

^{1}H NMR of 4-methyl-2-(1-*p*-tolylvinyl)phenol (**2c**)

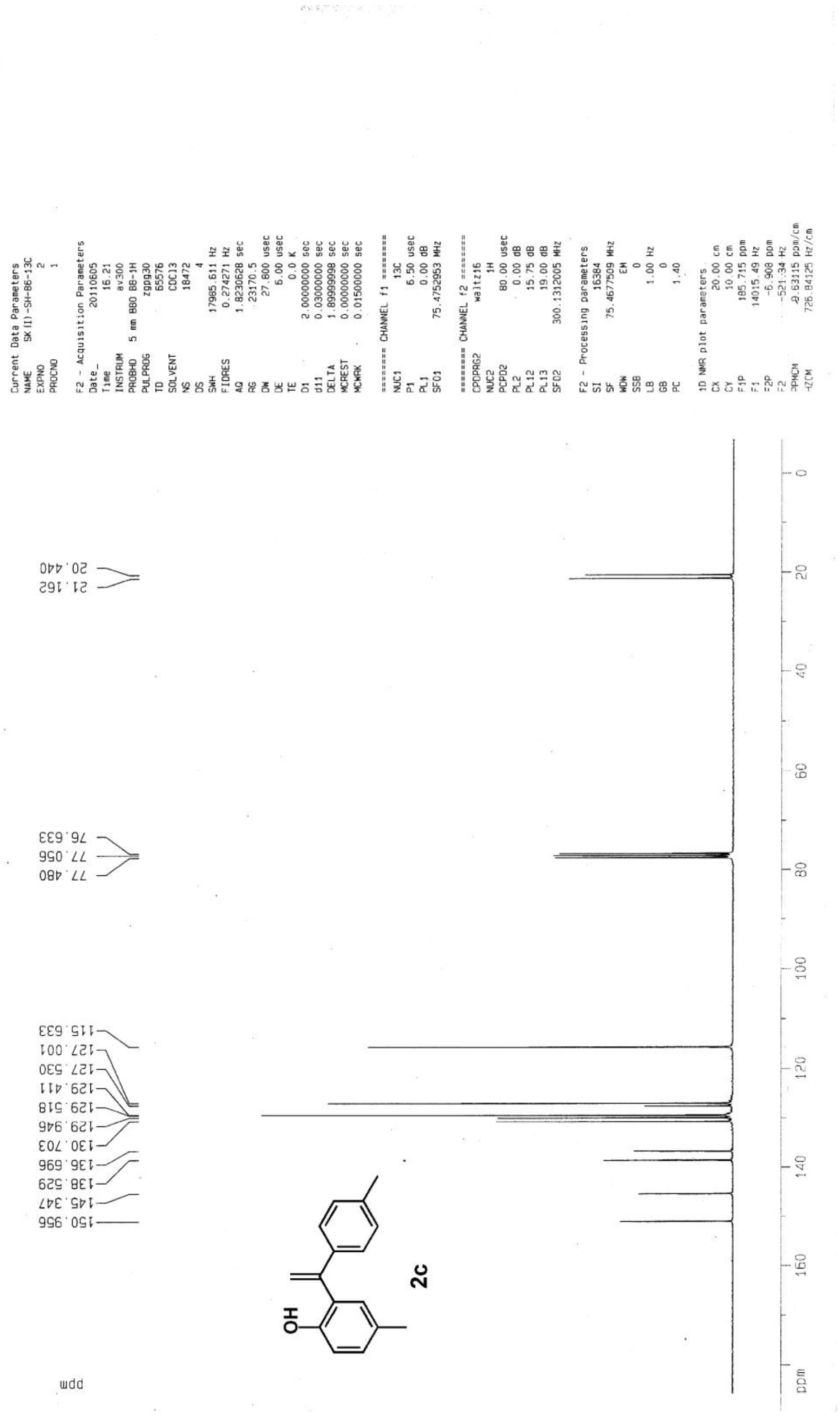

^{13}C NMR of 4-methyl-2-(1-*p*-tolylvinyl)phenol (**2c**)

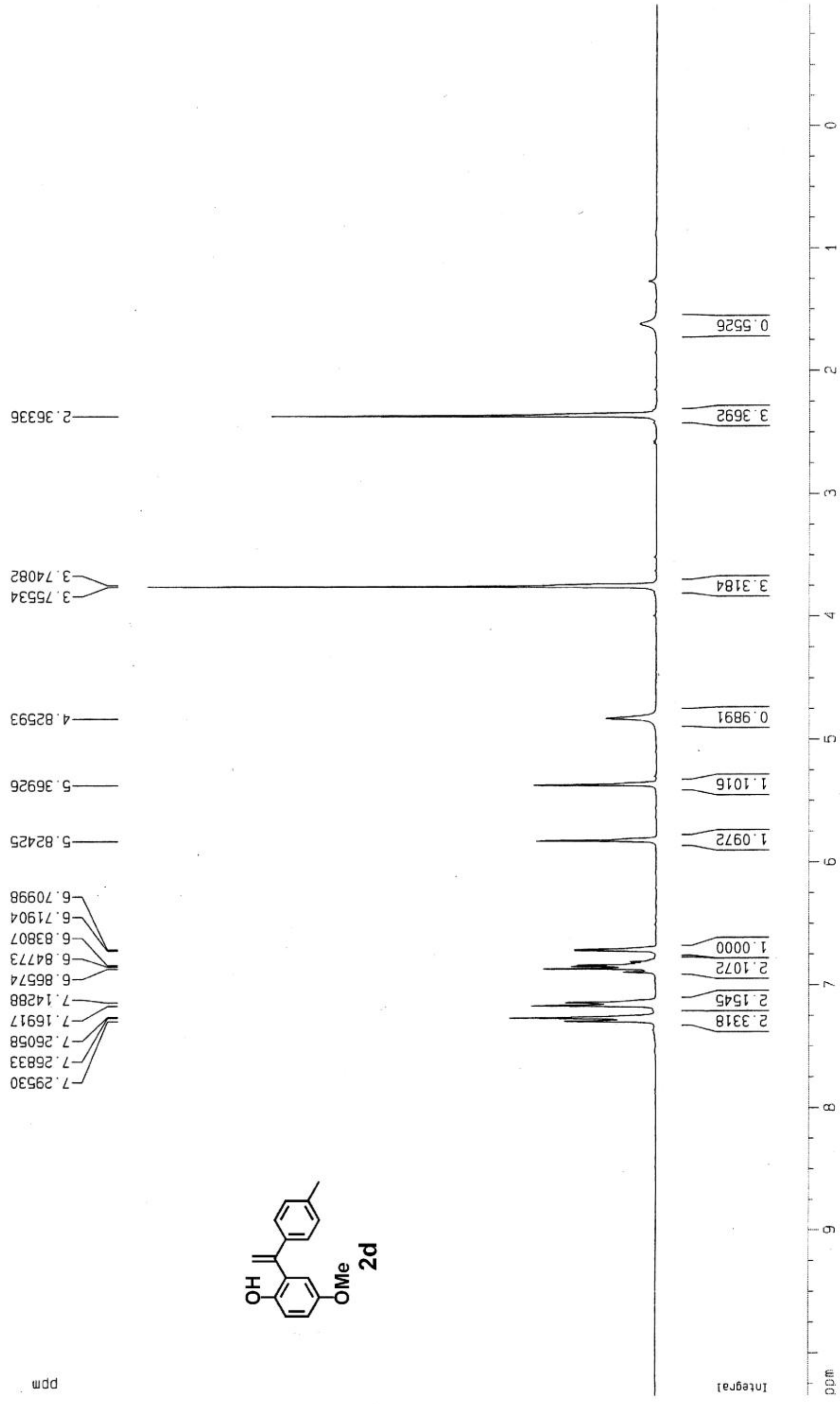

^{1}H NMR of 4-methoxy-2-(1-*p*-tolylvinyl)phenol (**2d**)

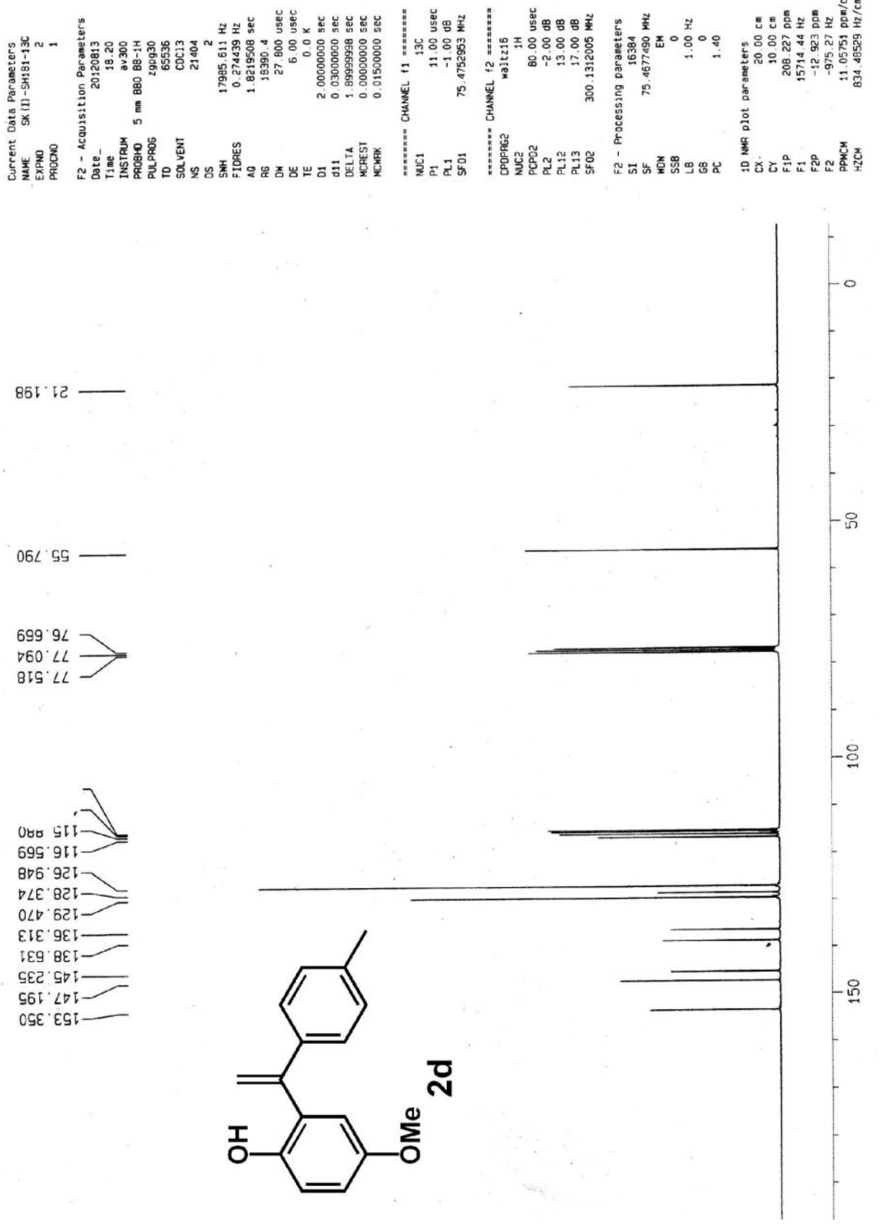

^{13}C NMR of 4-methoxy-2-(1-*p*-tolylvinyl)phenol (**2d**)
